# Mechanochemical synthesis of fluoride-ion conducting glass and glass–ceramic in ZrF_4_–BaF_2_ binary system

**DOI:** 10.1038/s41598-024-59040-4

**Published:** 2024-04-16

**Authors:** Kota Motohashi, Hiroshi Higuchi, Hiroshi Nakajima, Shigeo Mori, Atsushi Sakuda, Akitoshi Hayashi

**Affiliations:** 1 Department of Applied Chemistry, Graduate School of Engineering, Osaka Metropolitan University, 1-1 Gakuen-cho, Naka-ku, Sakai, Osaka 599-8531 Japan; 2 Department of Materials Science, Graduate School of Engineering, Osaka Metropolitan University, 1-1 Gakuen-Cho, Naka-Ku, Sakai, Osaka 599-8531 Japan

**Keywords:** Batteries, Batteries

## Abstract

Fluoride glasses in the binary system ZrF_4_–BaF_2_ were prepared via mechanochemical treatment. The glass-forming region of the ZrF_4_–BaF_2_ system obtained using the mechanochemical method was wider than that obtained using the conventional melt-quenching method. The glass–ceramic 60ZrF_4_·40BaF_2_ (mol%) sample was obtained by heat treatment and shows a higher conductivity of 1.2 × 10^–6^ S cm^−1^ at 200 °C than the pristine glass. This study revealed that mechanochemical treatment was effective for the synthesis of fluoride glasses.

## Introduction

Fluoride glasses have been widely investigated as materials for fiber optics^1,2^. Goldschmidt first reported BeF_2_ as a fluoride glass. BeF_2_ is the only fluoride that can form glass by itself. However, its extreme toxicity and high hygroscopicity render it an unsuitable device material^3^. In addition to fluoroberyllate glass, Poulain discovered fluorozirconate glass^4,5^. ZBLAN is a fluorozirconate glass that refers to the system of ZrF_4_–BaF_2_–LaF_3_–AlF_3_–NaF and is widely known as a practical material for optical fiber applications^6,7^. Fluoride glasses have been reported to exhibit optical and ionic conduction properties^8–12^. The latter property is expected to be suitable for solid electrolytes for all-solid-state fluoride-ion batteries and oxygen sensors, which have been the focus of much attention^13–17^. Although fluoride glasses are key materials used in optical and electrochemical devices, the synthesis of fluoride glasses is more difficult than that of oxide glasses because fluoride molten reacts with atmospheric oxygen and crucibles containing Al_2_O_3_ and SiO_2_. Thus, fluoride glasses basically require synthesis in an inert gas atmosphere in a glassy carbon crucible^18^. Furthermore, fluorine gas is generated during melting, which shifts the composition. NH_4_F·HF is extensively used as a fluorine source and an additive in fluoride mixtures^18^.

The mechanochemical (MC) method using a planetary ball mill is useful for solving these problems. The MC method is effective for compounds containing highly volatile elements because it uses a sealed container^19^. Sulfide lithium or sodium ion-conducting glasses are synthesized using the MC method^20,21^. Compared to the melt-quenching method, the MC method can be used to prepare glass with a wider composition^22^. Furthermore, in cation conductors, it is known that glass–ceramics prepared by heat treatment of glass have higher conductivity than glass when high ionic conducting phase forms^23,24^.

In this study, we applied the MC method to synthesize fluoride glasses, followed by the preparation of fluoride glass–ceramics by heat-treating the glasses. ZrF_4_–BaF_2_ glasses with fluoride-ion conductivity, which were previously prepared by the melt-quenching method, were selected as the model material^25^. Their structures, ionic conductivities, and thermal behaviors were investigated using X-ray diffraction (XRD), Raman spectroscopy, transmission electron microscopy (TEM), AC electrochemical impedance spectroscopy, and differential thermal analysis (DTA).

## Experimental

### Sample preparation

ZrF_4_ (99.9%, Strem Chem. Inc., USA) and BaF_2_ (99.9%, Kojundo Chem. Lab. Co. Ltd., Japan) were used to prepare the (100 − *x*)ZrF_4_·*x*BaF_2_ samples (*x* = 10, 15, 40, 45, and 50 mol%). These reagents were mixed in appropriate stoichiometric ratios in an Ar atmosphere. Samples were prepared by a mechanochemical (MC) method using a planetary ball mill apparatus (Pulversette 7, Fritsch Japan Co. Ltd., Japan) with zirconia pots (volume: 45 ml) and zirconia balls (diameter: 5 mm, mass: 75 g). The total mass of the starting material in each pot was 0.5 g, and the rotational speed and milling duration were 510 rpm and 50 h, respectively. To obtain the glass–ceramic sample, the 60ZrF_4_·40BaF_2_ (mol%) sample prepared by the MC method was heated at 235 °C for 2 h under Ar flow.

### Characterization

The obtained powders were characterized by XRD with Cu-Kα radiation (Smart Lab, Rigaku, Japan) and Raman spectroscopy with a 325 nm He–Cd laser (LabRAM HR800, Horiba Ltd., Japan).

The densities of the compact samples (*d*_1_) were calculated from the weights and volumes of the pellets, and those of the powders (*d*_2_) were measured using a gas pycnometer (AccuPyc II 1340, Shimadzu, Japan). The relative density was defined as *d*_1_ / *d*_2_.

The structure of the pellet cross-section was observed using scanning electron microscopy (SEM; JSM-6610A, JEOL, Japan).

A DTA curve was obtained for the powder sample in an Al pan under a N_2_ atmosphere using a thermal analyzer (Thermo-plus 8110, Rigaku, Japan). The heating rate was 10 °C min^−1^ from room temperature to 500 °C.

TEM was performed using a JEM-2100Plus instrument at an acceleration voltage of 200 kV (JEOL, Japan). High-resolution TEM (HR-TEM) images were obtained using a high-speed camera (OneView, Gatan) to minimize electron damage. The synthesized powders were dispersed on carbon grids in an Ar atmosphere in a glove box. The specimens were transferred without exposure to air using a vacuum transfer holder (Mel-Build Co.).

### Electrochemical impedance spectroscopy

The obtained powder samples were pelletized at 360 MPa via uniaxial pressing. Au thin-film electrodes were sputtered on both sides of the dense pellets. Electrical conductivities were measured by two-terminal AC electrochemical impedance spectroscopy in an Ar atmosphere from 30 to 300 °C with an amplitude voltage of 90 mV and a frequency of 1.0 × 10^7^–1.0 × 10^−1^ Hz using an impedance analyzer (Solartron 1260, Solartron Metrology, UK).

## Results and discussion

**Figure 1:**
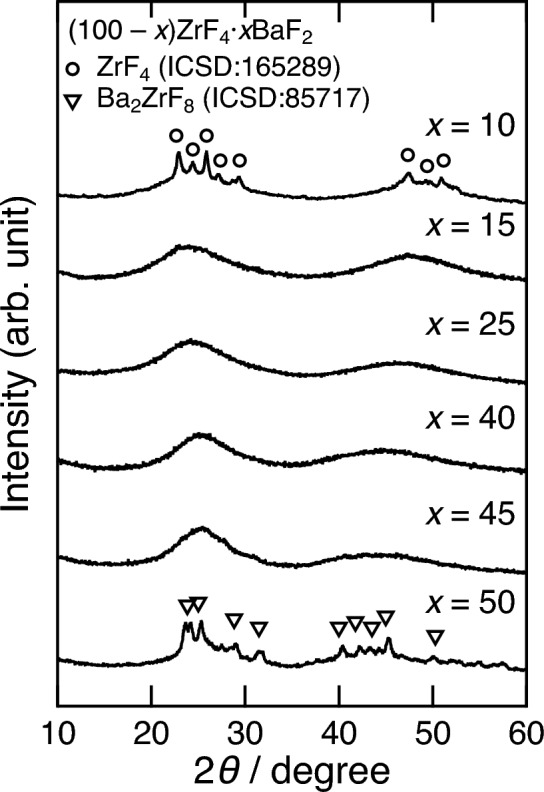
XRD patterns of the (100 − *x*)ZrF_4_·*x*BaF_2_ (*x* = 10–50 mol%) samples.

Figure 1 shows the XRD patterns of the (100 − *x*)ZrF_4_·*x*BaF_2_ (*x* = 10–50 mol%) samples prepared using the mechanochemical (MC) method. The XRD patterns of the *x* = 15, 25, 40, and 45 samples were halo-pattern, suggesting that the samples were amorphous. The *x* = 10 sample contained a fraction of ZrF_4_ reagent. Diffraction peaks attributable to Ba_2_ZrF_8_ (ICSD:85,717) were observed for the *x* = 50 sample.

**Figure 2:**
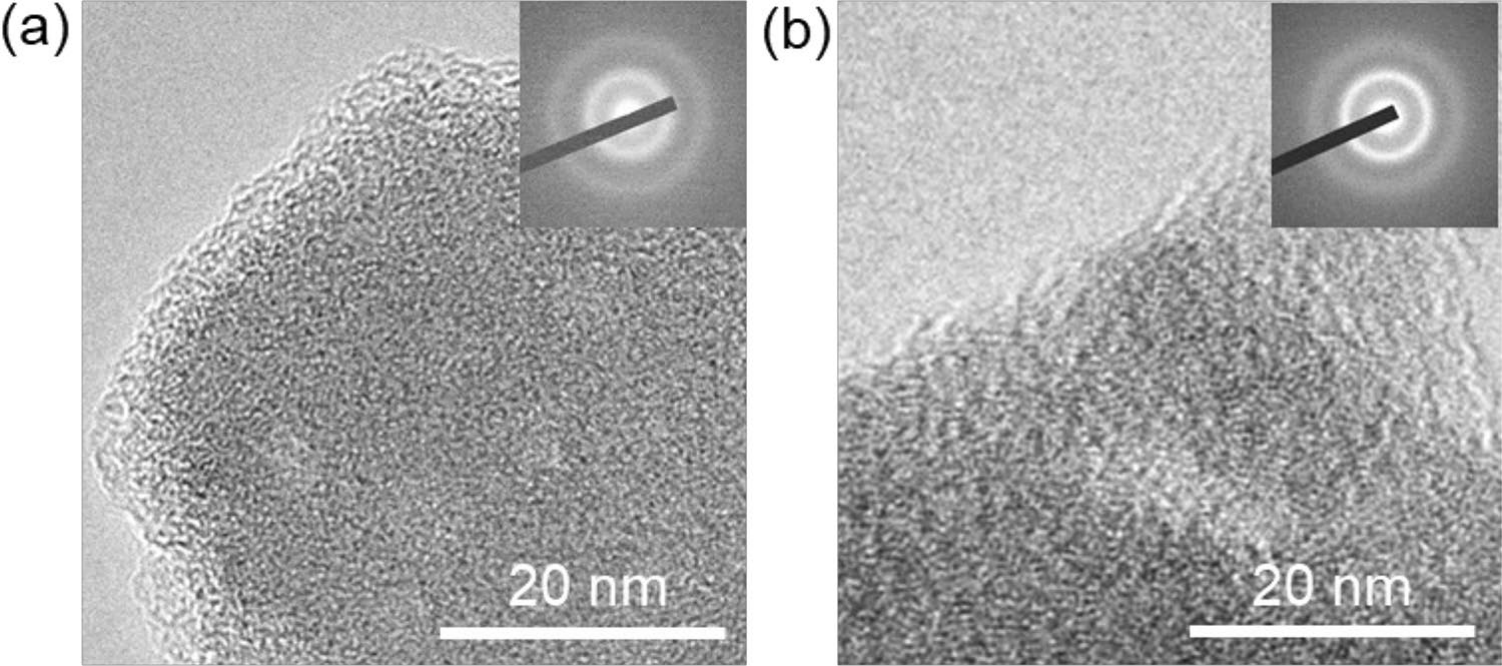
High-resolution TEM images and ED patterns of the (**a**) 85ZrF_4_·15BaF_2_ and (**b**) 55ZrF_4_·45BaF_2_ samples.

To investigate the morphology of the (100 − *x*)ZrF_4_·*x*BaF_2_ (*x* = 15 and 45) samples, TEM observations were carried out. Figure 2 shows HR-TEM images and electron diffraction (ED) patterns of the (100 − *x*)ZrF_4_·*x*BaF_2_ (*x* = 15 and 45) samples. The HR-TEM images depict typical amorphous contrast without periodic crystalline lattices. Accordingly, the halo patterns without crystalline spots were observed in the ED patterns obtained from the same region. Thus, an amorphous phase was mainly formed in the range of 15 ≤ *x* ≤ 45. The glass-forming region of the (100 − *x*)ZrF_4_·*x*BaF_2_ system by the MC method is wider than that of the reported melt-quenching method (25 ≤ *x* ≤ 45)^25^. The ZrF_4_–BaF_2_ fluoride-glasses were prepared with a stoichiometric ratio using MC method. This technique could also be effective for the synthesis of fluoride glasses in other systems.

**Figure 3:**
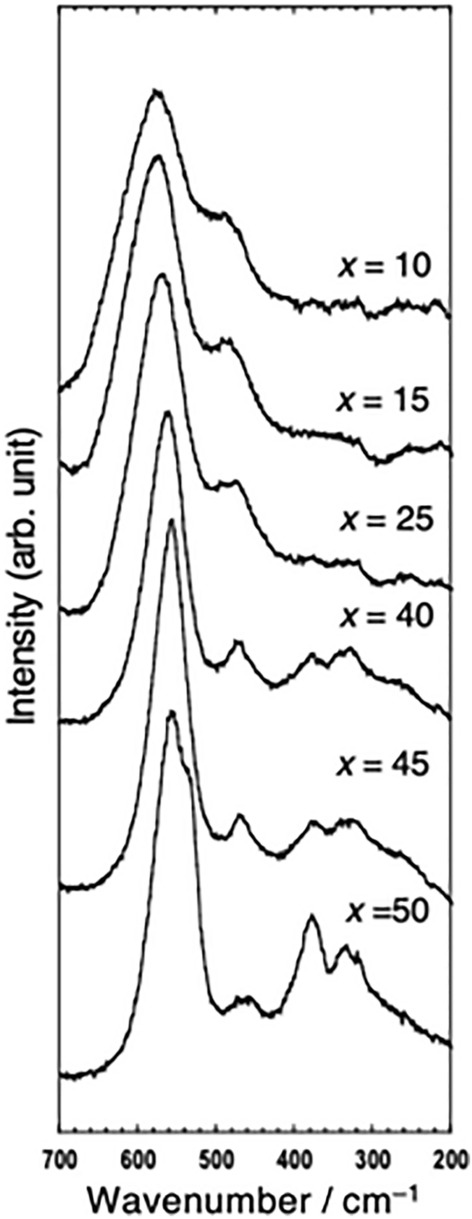
Raman spectra of the (100 − *x*)ZrF_4_·*x*BaF_2_ (*x* = 10–50 mol%) samples.

The local structures of the (100 − *x*)ZrF_4_·*x*BaF_2_ (*x* = 10–50 mol%) samples were analyzed using Raman spectroscopy. Figure 3 shows the Raman spectra of the (100 − *x*)ZrF_4_·*x*BaF_2_ (*x* = 10–50 mol%) samples prepared using the MC method. Bands at approximately 580 cm^−1^ and 480 cm^−1^ were observed and attributed to the Zr–F non-bridging symmetrical stretch vibration and the Zr–F bridging asymmetrical stretch vibration, respectively^26^. The intensity ratio of *I*_580_/*I*_480_ increased with increasing BaF_2_ content in the ZrF_4_–BaF_2_ system. This trend suggests that the proportion of non-bridging Zr–F increases.

**Figure 4:**
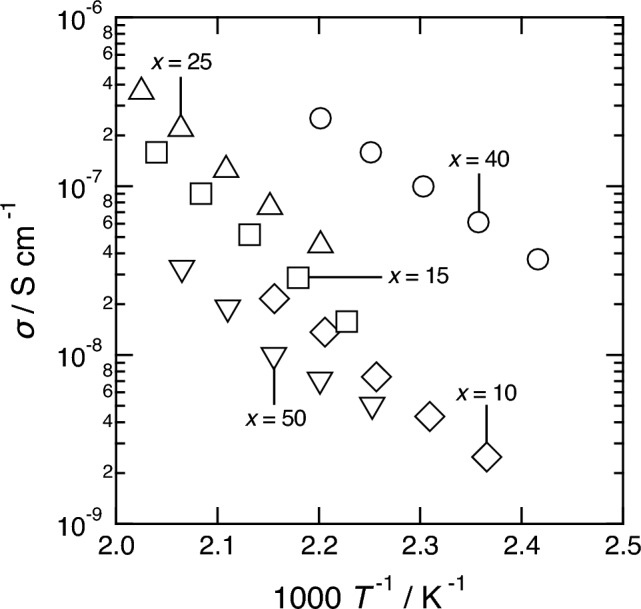
Temperature dependence of the ionic conductivities of the (100 − *x*)ZrF_4_·*x*BaF_2_ (*x* = 10–50 mol%) samples.

To measure the electrical conductivities of the samples, the prepared powders were pelletized at 360 MPa by uniaxial pressing. The densities of the compact pellets (*d*_1_) and powders (*d*_2_) and the relative densities of the prepared samples are listed in Table 1. The relative densities of all the samples were approximately 70%. SEM images of the cross sections of the pressed sample of 60ZrF_4_·40BaF_2_ are shown in Fig. S1. The pellets seemed dense as just pressed. The Nyquist plot obtained for the *x* = 40 sample at 181 °C is shown in Fig. S2. The Nyquist plot consists of a semicircle in the high-frequency region and a sharp spike in the low-frequency region, suggesting that the prepared sample is a typical ionic conductor. The total resistance of the sample was used to determine its conductivity. Figure 4 shows the temperature dependence of the ionic conductivities of the (100 − *x*)ZrF_4_·*x*BaF_2_ (*x* = 10–50 mol%) samples. The conductivities were enhanced with increasing BaF_2_ content, but reached a maximum at *x* = 40 and decreased with further increase in BaF_2_ content. The increase in conductivity is because of the increased Zr–F non-bridging content based on the Raman spectroscopy measurement results. In contrast, the decrease in the conductivity of the *x* = 50 sample was caused by precipitation of the Ba_2_ZrF_8_ crystal (Fig. 1).

**Table 1:** Densities of pellets (*d*_1_) and powder (*d*_2_), and relative densities (*d*_1_/*d*_2_) of the prepared (100 − *x*)ZrF_4_·*x*BaF_2_ samples.

*x* / mol%	*d*_1_ / g cm^–3^	*d*_2_ / g cm^–3^	Relative density / %
10	2.45	4.08	60
15	2.91	4.20	69
25	3.02	4.29	70
40	3.14	4.42	71
45	3.24	4.47	73
50	3.31	4.74	70

**Figure 5:**
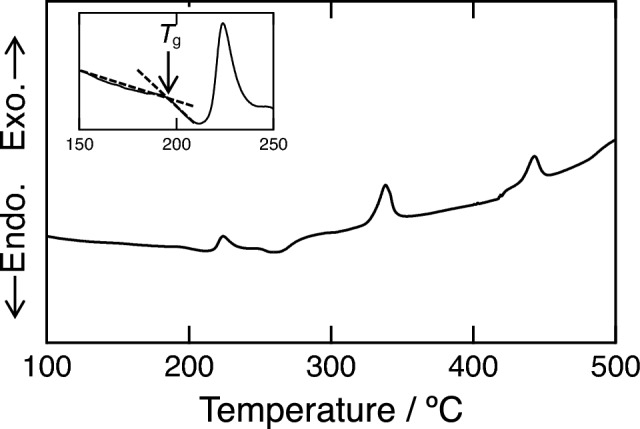
DTA curve of the 60ZrF_4_·40BaF_2_ sample. Inset shows an enlarged view of the glass transition temperature.

**Figure 6:**
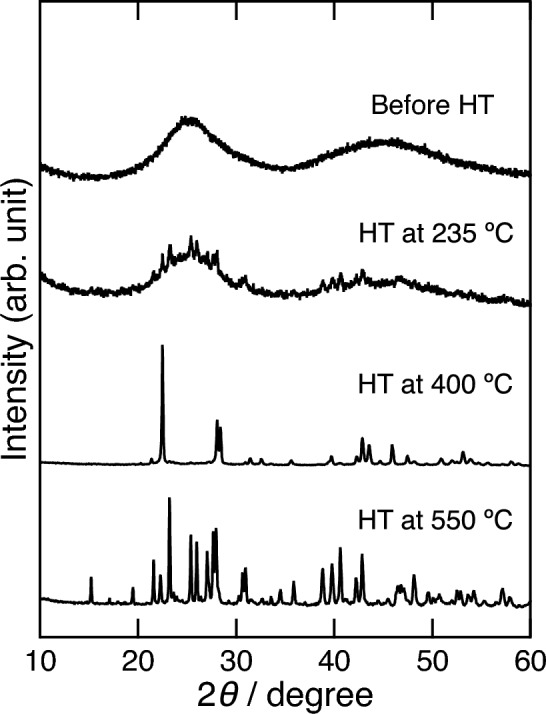
XRD patterns of the 60ZrF_4_·40BaF_2_ sample before and after heat treatment (HT).

To increase the ionic conductivity of the *x* = 40 sample, we prepared a glass–ceramic sample via heat treatment. The DTA curve of the 60ZrF_4_·40BaF_2_ sample prepared using the MC method is shown in Fig. 5. A baseline change attributable to the glass transition was observed at 193 °C. Exothermic peaks were also observed at 210, 330, and 440 °C. The exothermic peak at 210 °C is not observed in the reported sample prepared using the melt-quenching method^27^. According to previous reports, the exothermic peaks at 330 and 440 °C correspond to the crystallization temperature from glass to β-BaZrF_6_ and transformation temperature from β-BaZrF_6_ to α-BaZrF_6_, respectively^27^. The sample was heated at 235, 400, and 550 °C based on the exothermic peak temperatures in the DTA curve (Fig. 5). The XRD patterns of the heated samples are shown in Fig. 6. After heating at 235 °C, the sample exhibited a halo-pattern and contained small diffraction patterns of α-BaZrF_6_ crystal. This is because a high-temperature phase tends to precipitate as the primary phase in crystallization from a glass phase^28^. The XRD pattern of the sample heated at 400 and 550 °C could be indexed with β-BaZrF_6_ and α-BaZrF_6_, respectively.

**Figure 7:**
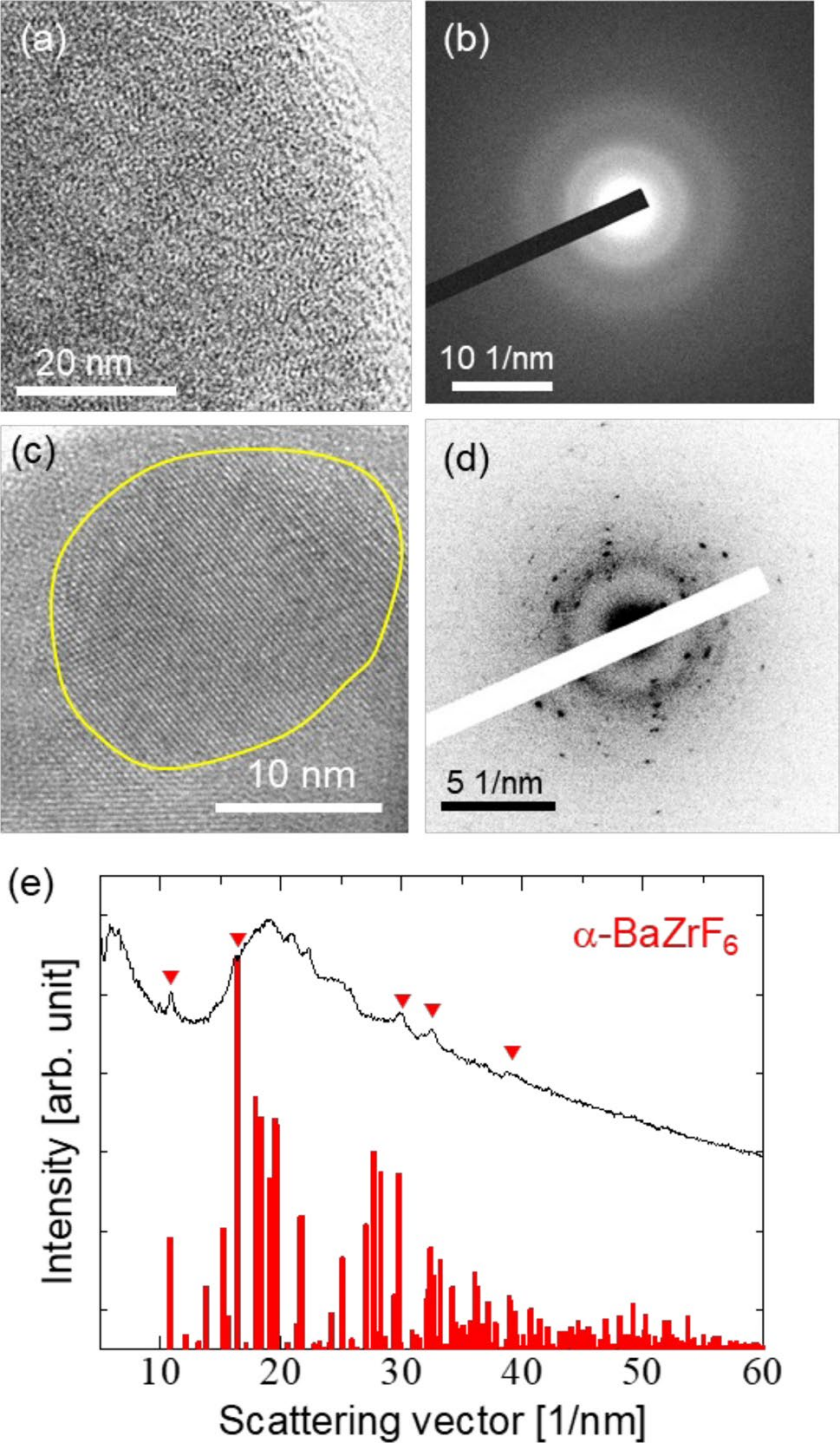
(**a**, **c**) High-resolution TEM image, (**b**, **d**) ED patterns, and (**e**) radial averaged intensity profile of the ED pattern of the 60ZrF_4_·40BaF_2_ samples (**a**, **b**) before and (**c**–**e**) after heat treatment at 235 °C.

Figure 7 shows the HR-TEM images and ED patterns of the 60ZrF_4_·40BaF_2_ samples before and after the heat treatment at 235 °C. No lattice fringes or Bragg spots were detected in the sample without heat treatment (Fig. 7a and b). These observations suggest that the sample without heat treatment was amorphous. Conversely, in the sample after heat treatment, the HR-TEM images show lattice fringes indicated by the circles in Fig. 7c, demonstrating that crystalline nanoparticles were formed after heat treatment. Crystalline spots and halo patterns are observed, as shown in Fig. 7d. Consistent with the XRD results, the ED spots are indexed to α-BaZrF_6_ (Fig. 7e), which is the high-temperature phase. These results indicate that the heat-treated sample was composed of amorphous and crystal phase of α-BaZrF_6_.

**Figure 8:**
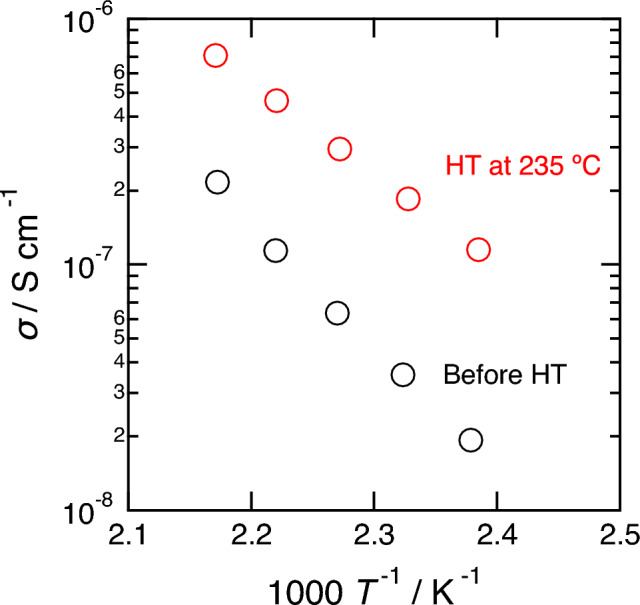
Temperature dependence of the ionic conductivities of the 60ZrF_4_·40BaF_2_ sample before and after heat treatment (HT) at 235 °C.

Figure 8 shows temperature dependance of the ionic conductivity of 60ZrF_4_·40BaF_2_ heated at 235 °C. The 60ZrF_4_·40BaF_2_ sample with heat treatment at 235 °C achieved 3 times higher conductivity than that without heat treatment at 200 °C (1.2 × 10^−6^ S cm^−1^). The activation energy of the heated sample (0.74 eV) was lower than that of the non-heated sample. The reported ionic conductivities at 200 °C and activation energies of α-BaZrF_6_ and β-BaZrF_6_ are 1.9 × 10^−9^ and 3.7 × 10^−8^ S cm^−1^ and 1.02 and 0.89 eV, respectively^25^. If α- and/or β-BaZrF_6_ crystalline phases appeared in the glass phase, the conductivity decreased. However, the conductivity increased owing to crystal precipitation. This is believed to be a unique phenomenon in glass–ceramic solid electrolytes; for example, vacancies are introduced during the crystallization process, as in Na_3_PS_4_ glass-ceramics^29^.

## Conclusion

We successfully prepared ZrF_4_–BaF_2_ glass using the MC method. The glass-forming region in the ZrF_4_–BaF_2_ binary system was expanded using the MC method compared with the melt-quenching method. 60ZrF_4_·40BaF_2_ glass–ceramics prepared by heat treatment were found to exhibit higher ionic conductivity than those without heat treatment. This study revealed that the MC method is effective for the synthesis of fluoride-ion glass. In addition, this study demonstrates that fluoride glass and glass–ceramics are a promising material group for noble fluoride-ion-conducting materials.

### Supplementary Information

Supplementary Figures.

## Data Availability

Data underlying the results presented in this paper are available from Dr. Kota Motohashi (kota.motohashi@omu.ac.jp) upon reasonable request.
