# Supplementary material for: Mechanochemical synthesis of fluoride-ion conducting glass and glass–ceramic in ZrF4–BaF2 binary system
Source: Sci Rep. 2024 Apr 16;14:8808. doi: 10.1038/s41598-024-59040-4 (PMC11021453; doi:10.1038/s41598-024-59040-4)
Supplement: Supplementary file 1 — Supplementary Figures. [file 41598_2024_59040_MOESM1_ESM.pdf]

Supplementary information (SI)

Mechanochemical Synthesis of Fluoride-ion Conducting Glass and Glass-ceramic in the ZrF_4–BaF_2 Binary System

K. Motohashi[1], H. Higuchi[1], H. Nakajima[2], S. Mori[2], A. Sakuda[1], A. Hayashi[1]*

[1] Department of Applied Chemistry, Graduate School of Engineering,

Osaka Metropolitan University, 1-1 Gakuen-cho, Naka-ku, Sakai, Osaka 599-8531, Japan.

[2] Department of Materials Science, Graduate School of Engineering,

Osaka Metropolitan University, 1-1 Gakuen-cho, Naka-ku, Sakai, Osaka 599-8531, Japan.

*Corresponding author: Akitoshi Hayashi

Department of Applied Chemistry, Graduate School of Engineering,

Osaka Metropolitan University, 1-1 Gakuen-cho, Naka-ku, Sakai, Osaka 599-8531, Japan.

Tel.: +81-72-254-9331

Fax.: +81-72-254-9910

E-mail: akitoshihayashi@omu.ac.jp

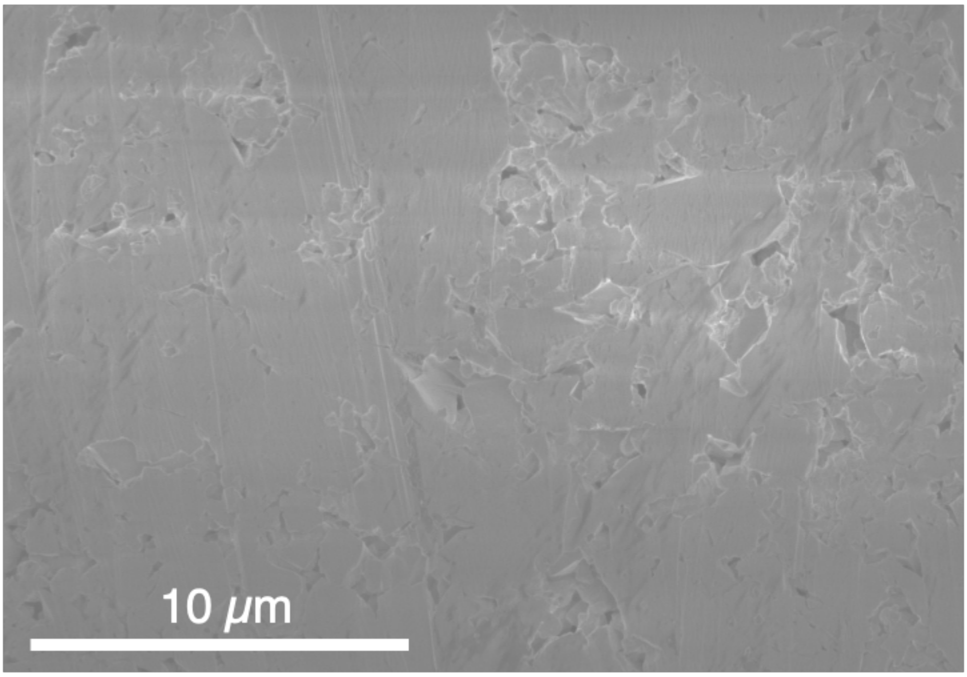

Figure S1. SEM image of the cross section of the pellet of $60ZrF_4 \cdot 40BaF_2$.

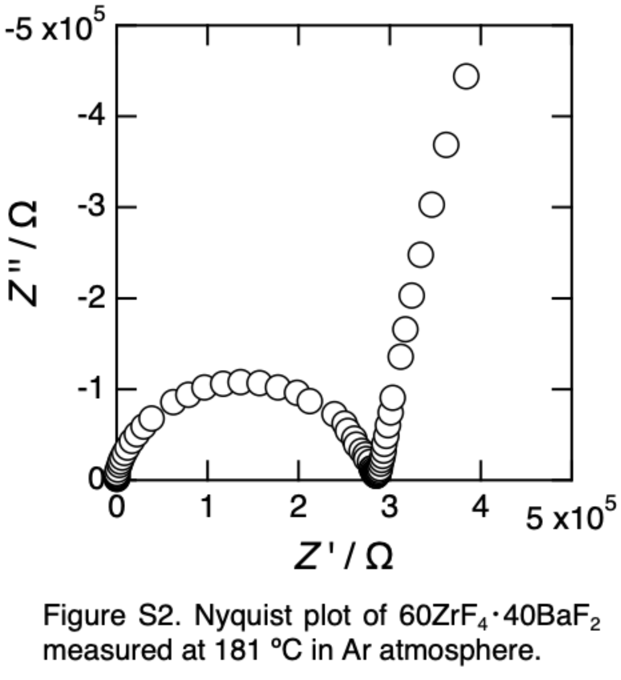

Figure S2. Nyquist plot of $60ZrF_4 \cdot 40BaF_2$ measured at 181 ºC in Ar atmosphere.
